# Discovery of a polyomavirus in European badgers (*Meles meles*) and the evolution of host range in the family *Polyomaviridae*

**DOI:** 10.1099/vir.0.000071

**Published:** 2015-06

**Authors:** Sarah C. Hill, Aisling A. Murphy, Matthew Cotten, Anne L. Palser, Phillip Benson, Sandrine Lesellier, Eamonn Gormley, Céline Richomme, Sylvia Grierson, Deirdre Ni Bhuachalla, Mark Chambers, Paul Kellam, María-Laura Boschiroli, Bernhard Ehlers, Michael A. Jarvis, Oliver G. Pybus

**Affiliations:** ^1^​ Department of Zoology, University of Oxford, UK; ^2^​ School of Biomedical and Healthcare Sciences, Plymouth University, UK; ^3^​ Wellcome Trust Sanger Institute, UK; ^4^​ Bacteriology Department, Animal and Plant Health Agency, UK; ^5^​ School of Veterinary Medicine, University College Dublin (UCD), Ireland; ^6^​ ANSES, Nancy Laboratory for Rabies and Wildlife, France; ^7^​ Department of Virology, Animal and Plant Health Agency, UK; ^8^​ School of Veterinary Medicine, University of Surrey, UK; ^9^​ MRC/UCL Centre for Medical Molecular Virology, University College London, UK; ^10^​ University Paris-Est, ANSES, Laboratory for Animal Health, Bovine Tuberculosis National Reference Laboratory, France; ^11^​ Robert Koch Institute, Division 12 ‘Measles, Mumps, Rubella and Viruses Affecting Immunocompromised Patients’, Germany

## Abstract

Polyomaviruses infect a diverse range of mammalian and avian hosts, and are associated with a variety of symptoms. However, it is unknown whether the viruses are found in all mammalian families and the evolutionary history of the polyomaviruses is still unclear. Here, we report the discovery of a novel polyomavirus in the European badger (*Meles meles*), which to our knowledge represents the first polyomavirus to be characterized in the family Mustelidae, and within a European carnivoran. Although the virus was discovered serendipitously in the supernatant of a cell culture inoculated with badger material, we subsequently confirmed its presence in wild badgers. The European badger polyomavirus was tentatively named Meles meles polyomavirus 1 (MmelPyV1). The genome is 5187 bp long and encodes proteins typical of polyomaviruses. Phylogenetic analyses including all known polyomavirus genomes consistently group MmelPyV1 with California sea lion polyomavirus 1 across all regions of the genome. Further evolutionary analyses revealed phylogenetic discordance amongst polyomavirus genome regions, possibly arising from evolutionary rate heterogeneity, and a complex association between polyomavirus phylogeny and host taxonomic groups.

## Introduction

Viruses of the family *Polyomaviridae* are small, non-enveloped icosahedral viruses, comprising ~70 putative species from three proposed genera (Imperiale & Major, 2007; Johne *et al.*, 2011). Polyomaviruses have been found in many different avian and mammalian hosts, including rodents, birds, bats, humans, non-human primates, carnivorans, elephants, dolphins, horses and Artiodactyla. In human populations, polyomavirus seroprevalence can reach up to 90 % and most infections are asymptomatic (Van Ghelue *et al.*, 2012). However, in immunocompromised individuals infection can cause an array of symptoms, including Merkel cell carcinoma, kidney disease and progressive multifocal leukoencephalopathy (Imperiale & Major, 2007). Although renal and respiratory diseases, tumours, and wasting have been reported in some other animals, the consequences of polyomavirus infection in many non-human species remain to be determined.

**Fig. 1.**
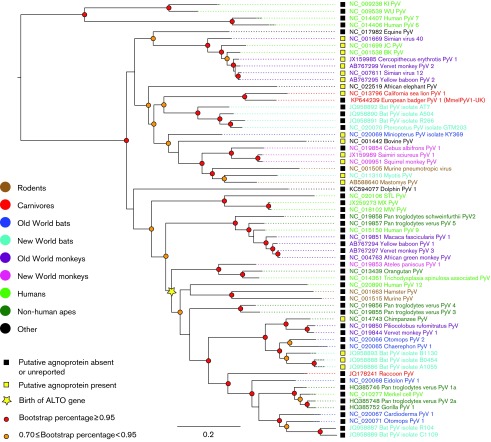
Maximum-likelihood phylogeny of all known polyomavirus (PyV) species or putative species, including MmelPyV1, estimated from the ‘genome-wide’ alignment. Viral taxa are coloured according to host species and bootstrap support scores are indicated using coloured circles (as indicated in the key). Scale bar represents expected number of substitutions per site (first and second codon positions only).

All identified polyomaviruses share a similar genome organization and virion structure. Polyomaviruses typically exhibit a 40–45 nm diameter non-enveloped icosahedral capsid, composed of 72 monomers (Baker *et al.*, 1989; Imperiale & Major, 2007). The capsid encloses a single, circular dsDNA genome of ~5000 bp (Johne *et al.*, 2011). All polyomavirus genomes are composed of early and late regions, and regulatory regions called the non-coding control region (NCCR) (Imperiale & Major, 2007). The NCCR contains the origin of replication, transcription factor binding sites, promoters and enhancers. Transcription from the NCCR is bidirectional, producing mRNA encoding either early proteins or late proteins (Johne *et al.*, 2011). All polyomaviruses produce at least two early mRNA products encoding large and small tumour antigens (LT-Ag and St-Ag, respectively) (Imperiale & Major, 2007). These proteins are involved in viral genome replication and modulation of host cell conditions required for virus replication (Stevens *et al.*, 2013). The mouse and hamster polyomaviruses also encode a middle tumour antigen (MT-Ag) (Imperiale & Major, 2007). Recently, an alternate reading frame gene (called ALTO) overlapping the LT-Ag gene was identified in Merkel cell polyomavirus (MCPyV) and predicted to occur in polyomaviruses that are phylogenetically related to MCPyV (Carter *et al.*, 2013). Late polyomavirus mRNA transcripts encode capsid proteins (typically VP1, VP2 and VP3) (Imperiale & Major, 2007; Johne *et al.*, 2011). Several mammalian polyomaviruses (Fig. 1) also encode an agnoprotein or other short proteins between the NCCR and the VP2 ORF (Johne *et al.*, 2011). Agnoproteins are involved in the control of viral protein expression and have effects throughout the viral life cycle (Gerits & Moens, 2012).

Virus cross-species zoonotic transmission is a frequent cause of emerging epidemics in humans (e.g. influenza A viruses, Lassa virus, Ebola virus, severe acute respiratory syndrome coronavirus and Middle East respiratory syndrome coronavirus) (Parrish *et al.*, 2008). Understanding the evolutionary history of viral families can help define the propensity of viruses to switch host species. However, little is known about the evolutionary history of polyomaviruses or their capacity for host switching. In an early phylogenetic study (Shadan & Villarreal, 1993), the similarity of mammalian and polyomavirus evolutionary trees led to the suggestion that polyomaviruses had co-evolved with their host species. This idea evolved to incorporate a combination of host-switching and virus–host co-divergence events (notably, the basal split between avian and mammalian polyomaviruses) (Pérez-Losada *et al.*, 2006). However, recent statistical re-evaluation that incorporated novel polyomaviruses from a wider range of taxa has largely rejected the hypothesis of polyomavirus–host co-divergence (Krumbholz *et al.*, 2009; Warden & Lacey, 2012).

In the absence of evidence for co-divergence, alternative models have been proposed to explain the phylogenetic distribution of polyomavirus host species. In the first model, cross-species transmission of polyomaviruses is proposed to be a relatively common evolutionary event (Tao *et al.*, 2013). This hypothesis has consequences for human and animal health, and raises the question of what factors determine the rate of virus transmission between species (Streicker *et al.*, 2010). In the second model, the polyomavirus phylogeny is proposed to result from recombination amongst polyomaviruses, which results in the appearance of host species switching. A recent study found support for recombination of several polyomaviruses, yet was unable to identify ancestors of the putative recombinant lineages with certainty (Tao *et al.*, 2013). In a third model, heterogeneity in the evolutionary rates of different genes amongst the polyomavirus lineages is used to explain their complex evolutionary history (Tao *et al.*, 2013).

Here, we report Meles meles polyomavirus 1 (MmelPyV1) as a novel polyomavirus present in wild European badger (*Meles meles*) populations and attempt to clarify the evolutionary history of the mammalian polyomaviruses. To our knowledge, this represents the first description of a polyomavirus in the family Mustelidae, expanding the host range of these viruses for which full-length sequences exist to 18 distinct mammalian families. To explore the evolutionary history of this novel virus and the mammalian polyomaviruses as a whole, we performed comprehensive phylogenetic analyses and analyses of viral recombination using whole-genome sequences from all available mammalian polyomavirus species.

## Results

### Virus discovery in cell culture

The badger polyomavirus was first discovered in a cell culture supernatant derived from tissues taken from a badger lung in Cornwall, England, in 1996 (Banks *et al.*, 2002). The original purpose of the culture was to maintain a badger herpesvirus, *mustelid herpesvirus 1* (MusHV-1), present in the original tissue sample (Banks *et al.*, 2002). The clarified supernatant of this cell culture had been stored at −80°C since 1997 (henceforth referred to as ‘1997 supernatant’). The 1997 supernatant was enriched using a sucrose cushion as part of an attempt to sequence the badger herpesvirus present. DNA was extracted from the sucrose-cushion-purified supernatant, amplified using random priming and sequenced using Illumina deep sequencing.

**Fig. 2.**
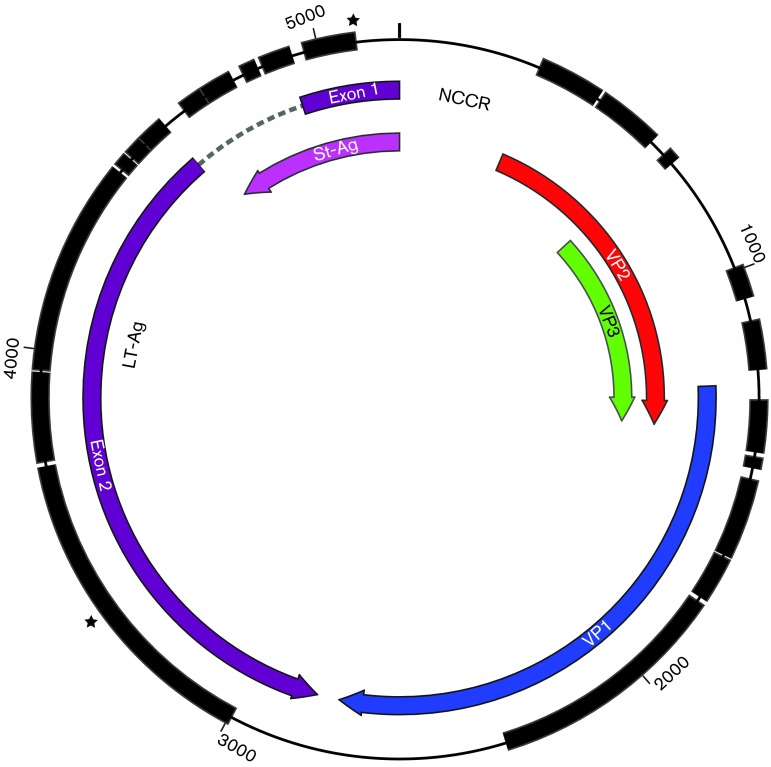
Genome map of MmelPyV1. Thick black blocks (outer circle) represent genomic regions that could be aligned across all mammalian polyomaviruses and were therefore included in the ‘genome-wide’ alignment. Coloured arrows represent ORFs. Early proteins are in purple; late proteins are in blue, red and green. Breakpoints used in recombination analysis are marked with two stars.

In addition to herpesvirus contigs (in preparation), two polyomavirus contigs of 4495 and 468 nt were identified, which appeared to encode a full-length circular genome (Fig. 2) (here denoted the MmelPyV1 UK isolate; GenBank accession number KP644239). Overlapping identical sequences of 110 nt were found at both ends of the molecules. Furthermore, additional reads were present in the short read sequence data that spanned both ends of the sequences. When the original MiSeq reads were mapped to the genome, 1278 reads (of 6.3 million) were identified with perfect homology to the genome. In alignable regions, nucleotide identity with any other polyomavirus genome was never more than 72 % and the closest known viral genome was that of California sea lion polyomavirus 1 (CSLPyV1). Under the definition of <81–84 % sequence identity suggested by Johne *et al.* (2011), the polyomavirus described here represents a new species and the species name ‘Meles meles polyomavirus 1’ (MmelPyV1) is proposed.

**Fig. 3.**
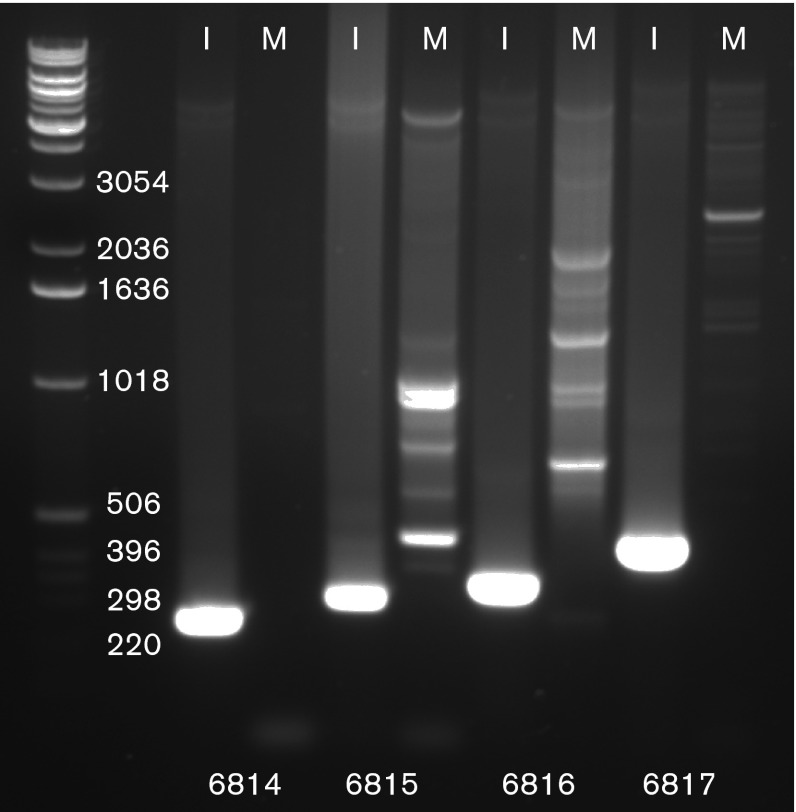
Gel electrophoresis of mink cell line NBL-7. Lanes: I, infected with UK isolate cell line; M, mock infected. Numbers below the lanes represent the primers used. Numbers on the left represent the ladder fragment size (bp). Controls not shown.

To determine that the polyomavirus was not a contaminant in the mink cell line used to culture the badger herpesvirus isolate, the mink cell line was tested for the presence of the virus. Primers designed from the MmelPyV1 genome generated strong PCR bands in a mink cell line sample that had been inoculated with material generated from the 1997 supernatant (Fig. 3) (see Methods). Indistinct or no bands were present in the negative control mock-infected mink cell line sample. This finding was consistent with the polyomavirus being derived from the original badger lung tissue and not the mink cell line or cell culture reagents.

### Virus discovery in wild badgers

Fetal calf serum, which was used here throughout cell culturing, is the source of at least one species of polyomavirus and other species have been isolated from bovine tissue (Parry *et al.*, 1983; Peretti *et al.*, 2015; Schuurman *et al.*, 1991; Zhang *et al.*, 2014). To establish conclusively that MmelPyV1 occurrs in badgers, uncultured samples from wild badgers in France were tested in an independent laboratory for the presence of the virus. Diagnostic PCRs targeting VP1 and the NCCR were performed on faecal samples and seven different tissues from 11 different badgers to determine prevalence. Eight badgers (73 %) showed evidence of polyomavirus infection. PCR results were positive at least once in all sample types, except faecal samples which were all negative (Tables S1 and S2, available in the online Supplementary Material). Overall, 21 of 87 (24 %) samples were positive for MmelPyV1. Samples from liver and lymph nodes exhibited the highest rate of positivity. Neither VP1 nor the NCCR showed any sequence variability across the wild badger samples. Long-distance nested PCRs were used on one sample to generate an amplified genome, which was then sequenced. This generated a further polyomavirus isolate, referred to here as the MmelPyV1 French isolate (GenBank accession number KP644238).

### Genome characterization

Full-length comparison of the French and UK isolates showed that they differ by 34 single nucleotide polymorphisms (SNPs) and one indel. Thirty of the 34 SNPs fell in coding regions, four of which were non-synonymous (three in VP1 and one in LT-Ag) (Ser1770Thr, Ile2520Val, Ala2557Gly and Ala3044Pro; nucleotide positions given relative to the MmelPyV1 UK isolate genome). (Comparison to the type species SV40 is inappropriate here due to sequence length variation between MmelPyV1 and SV40 in VP1.) The single indel occurred in the NCCR.

The divergence time of the UK and French isolates was estimated based on previously published rates of polyomavirus nucleotide substitution. Assuming a substitution rate in coding regions of 4.34×10^−5^ substitutions per site per year [95 % confidence interval (CI) 2.42–6.41×10^−5^ substitutions per site per year] (Firth *et al.*, 2010), our observation of 30 SNPs in 4791 bp of coding sequence suggests that the French and UK isolates diverged within the past few hundred years (mean 144 years ago; 95 % CI 97–260 years ago). This estimate should be considered tentative as it is based on an evolutionary rate for the BK polyomavirus (Firth *et al.*, 2010) and the rate for MmelPyV1 may be different.

**Fig. 4.**
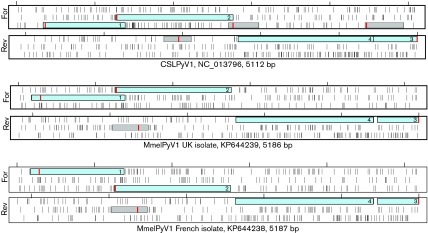
Conserved ORFs of the two MmelPyV1 isolates and their nearest relative (CSLPyV1). All six translation frames are shown; the three forward (For) and reverse (Rev) reading frames are shown in separate boxes. All ORFs >100 aa are shown as boxes, with the first methionine marked in red. Solid vertical lines indicate stop codons and tick marks indicate 1000 bp markers. Blue coloured ORFs represent probable protein-coding ORFs identifiable by numbering: 1, VP2 and VP3; 2, VP1; 3, St-Ag and exon 1 of LT-Ag; 4, exon 2 of LT-Ag. Details of GenBank accession numbers and genome length are also included.

The MmelPyV1 genome is a 5186 or 5187 bp circular molecule with a G+C content of 42.8 %. These values fall within the range for known mammalian polyomaviruses (length 4697–5722 bp; G+C content 36.4–47.3 %). The genome organization of MmelPyV1 was typical of the family *Polyomaviridae*, encoding ORFs from both strands of the genome separated by the NCCR (Fig. 2). ORFs encoding known viral proteins were conserved between the MmelPyV1 isolates and their closest relative, CSLPyV1 (Fig. 4). Proteins homologous to polyomavirus VP1, VP2, VP3, LT-Ag and St-Ag are present.

The LT-Ag sequence shares many features with other polyomaviruses, including a DnaJ domain (An *et al.*, 2012; Pipas, 1992) (including HPDKGG at aa 42–47; amino acid positions given relative to SV40), a Rb binding motif (An *et al.*, 2012; Pipas, 1992) (LRCDE at aa 103–107), ATPase motifs (Pipas, 1992) (GPINSGKT at aa 426–433 and GCVKVNLE at aa 503–510), a zinc finger motif (An *et al.*, 2012; Pipas, 1992) (CMDCLEEQIITHYKYH at aa 302–317), a TPPK motif (DeCaprio & Garcea, 2013) (TPPK at aa 124–127), a Bub-1 motif (An *et al.*, 2012) (WERWW at aa 91–95) and a Cr1 domain (Pipas, 1992) (LMQLL at aa 13–17). Other motifs identified in SV40 appear to be absent (Cul-7 FNXEX; Ehlers & Moens, 2014). The St-Ag has motifs similar to other polyomaviruses, including two conserved PP2A binding motifs (Ehlers & Moens, 2014) (CQRNVNPKCRCLMCRLKRKH at aa 103–122 and WGMCYCYSCYCQW at aa 135–148).

The NCCR contained three likely LT-Ag binding sites (An *et al.*, 2012; Pipas, 1992): one GAGGC at nt 275–279 and two reverse complement GCCTC at nt 77–81 and 84–88 (nucleotide positions relative to the UK isolate). Analyses using emboss (Rice *et al.*, 2000) showed no evidence of tandem repeats or palindromes in the NCCR.

Neither ALTO nor agnoprotein genes were identified in MmelPyV1. A 53 aa ORF was identified overprinting the LT-Ag exon 2 in a frameshift position, in a similar genome position to the previously proposed ALTO gene (Carter *et al.*, 2013). However, MmelPyV1 is phylogenetically distinct from the clade of ALTO-containing polyomaviruses (Fig. 1) and the ORF is considerably shorter than identified ALTO proteins (53 aa compared with 248–250 aa; Carter *et al.*, 2013). Consequently, this ORF is unlikely to represent an ALTO gene. After searching for ORFs that were (i) conserved between the two MmelPyV1 isolate genomes, (ii) >30 aa long (corresponding to the shortest proposed current agnoprotein; Misra *et al.*, 2009) and (iii) located in the forward direction upstream of the VP2 start codon, we found no evidence of an agnoprotein ORF. We discovered one ORF >100 aa long in MmelPyV1 (location indicated by grey box in Fig. 4). This ORF does not have sequence identity with any known polyomavirus proteins, and is not conserved between MmelPyV1 and the closely related CSLPyV1, so is unlikely to represent a protein-encoding ORF.

It is unknown whether MmelPyV1 causes disease. The badger from which the UK isolate was derived was malnourished, harboured MusHV-1 (a virus found in most badgers; King *et al.*, 2004), and exhibited non-specific symptoms of inflammation and lesions in the kidneys, liver and lungs (Banks *et al.*, 2002). The badgers from which the French isolate was obtained showed no obvious disease symptoms when examined by a veterinarian.

### Phylogenetic analysis of the family *Polyomaviridae*

To investigate the evolutionary history of the mammalian polyomaviruses, we constructed maximum-likelihood phylogenetic trees based on an alignment of 63 whole-genome polyomavirus sequences (obtained from GenBank in August 2014). In a maximum-likelihood phylogeny of polyomavirus genomes (Fig. 1), MmelPyV1 clustered with CSLPyV1 (Wellehan *et al.*, 2011). This result was supported by 100 % bootstrap support in all phylogenies we estimated. In the genome regions retained in the genome-wide alignment (but including all three codon positions) (Fig. 2), MmelPyV1 and CSLPyV1 shared 72 % nucleotide sequence identity. Based on a longer alignment of only the badger isolates and CSLPyV1 genomes across all coding regions, the estimated genetic *p* distance between the French and UK badger isolates was 0.006 substitutions per site, and between each MmelPyV1 isolate and the CSLPyV1 was 0.334 and 0.335 substitutions per site, respectively.

The maximum-likelihood phylogeny based on all known mammalian polyomavirus genomes comprises two well-supported clades: a smaller clade containing the KI, WU, HPyV6 and HPyV7 human polyomaviruses, and a larger clade containing a broader range of species (Fig. 1). MmelPyV1 and CSLPyV1 are clustered with monkey, bat, ape, rodent and dolphin viruses, amongst other hosts. This cluster is distinct from the Almipolyomavirus group that is defined by the presence of the ALTO gene (Carter *et al.*, 2013) (indicated by a yellow star in Fig. 1).

**Fig. 5.**
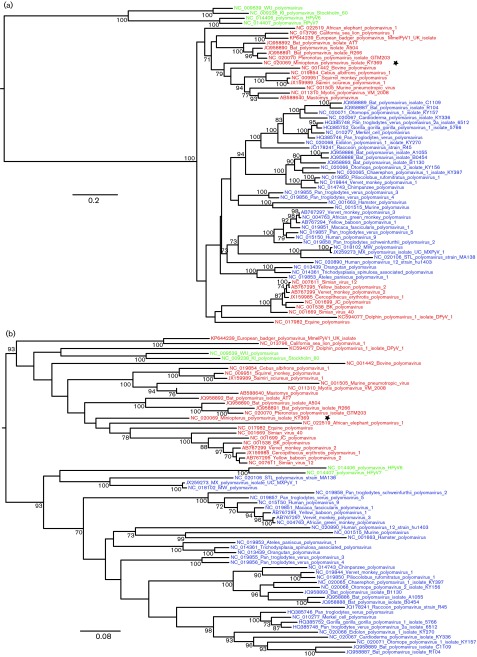
Maximum-likelihood phylogenies produced from different regions of the genome. (a) Partition A, comprising the late region plus a short region of LT-Ag exon 2. (b) Partition B, comprising the majority of the early region. Red and blue coloured branches indicate the major two clades as identified in the genome-wide alignment (Fig. 1). Green branches indicate human polyomaviruses within inconsistent phylogenetic locations. The *Miniopterus* polyomavirus is marked with a star to indicate identification in Recco as a further possible recombinant. Scale bar represents expected number of substitutions per site (first and second codon positions only).

The division into these two clades is not preserved when different parts of the genome are considered separately. Phylogenies estimated from overlapping 600 bp partitions of the genome-wide alignment (not shown) show that the WU, KI, HPyV6 and HPyV7 polyomaviruses, which form a strongly supported monophyletic clade in Fig. 1, are not consistently placed together when different subgenomic regions are analysed. Such phylogenetic incongruence may indicate that these taxa have recombined in their evolutionary history. Analysis using Recco indicated statistical support for recombination of HPyV6, HPyV7, WU and KI, and also for the *Miniopterus* polyomavirus (small black star in Fig. 5) (alignment *P* values <0.001). Recco also reported recombination in the equine polyomavirus lineage, although this was less well supported (*P* = 0.026).

The putative breakpoints in the circular genomes of WU, KI, HPyV6 and HPyV7 were identified as occurring in the NCCR and towards the 3′ end of the LT-Ag exon 2 (breakpoints marked on Fig. 2 with black stars). These breakpoint positions were used to define two partitions of the genome-wide alignment: (i) partition A, comprising the late region (~1000 nt) plus a short region of the LT-Ag exon 2 (~360 nt), and (ii) partition B, comprising the majority of the early region (~1000 nt of the genome-wide alignment). The phylogeny estimated from partition A (Fig. 5a) shows the WU, KI, HPyV6 and HPyV7 polyomaviruses to be a well-supported monophyletic cluster, placed as a sister group to the remainder of the mammalian polyomaviruses. The phylogeny estimated from partition B (Fig. 5b) does not support this cluster. Instead, WU and KI group together in a separate clade (red in Fig. 5b), and HPyV6 and HPyV7 group together in a separate clade (blue in Fig. 5b), in both instances with a bootstrap support of 93 %.

Visual inspection of the mammalian polyomavirus phylogenies indicates that viruses isolated from the same host family or order are not always clustered together, yet closely related virus species are often from similar hosts (Fig. 1). The program BaTS (Parker *et al.*, 2008) was used to quantify the statistical support for an association between the virus phylogeny and host species taxonomy. Host species were assigned to categories in three different ways (Table S3). In each case there was a significant correlation between host taxonomic category and viral phylogeny (Table 1). Thus, polyomaviruses from related hosts are grouped together in the viral phylogenetic tree more often than expected by chance (Table 1).

**Table 1.** Results of the BaTS tests for association of virus phylogeny with host species taxonomy

Host species grouping (no. of categories)	Mean association index	Mean parsimony score	*P*
Mammalian Order (8)	0.47	13.79	<0.01
Mammalian Family (19)	1.69	26.00	<0.01
Modified Order grouping* (10)	1.44	26.74	<0.01

* Host species are grouped according to mammalian order, except bats (split into Old/New World bats), primates (split into Old/New World monkeys and apes) and ungulates (grouped into a single category). See Table S3 for further details.

## Discussion

Under the species definition proposed by Johne *et al.* (2011), the polyomavirus reported here represents a novel species and we suggest the name ‘Meles meles polyomavirus 1’. To our knowledge, MmelPyV1 represents the first complete polyomavirus species found in the family Mustelidae. A metagenomic analysis of ferret faeces conducted by Smits *et al.* (2013) detected DNA fragments with sequence similarity to polyomaviruses in two ferrets, hinting that polyomaviruses may occur in mustelids (Smits *et al.*, 2013) – a hypothesis that has been confirmed here. Following the discoveries of the California sea lion and raccoon polyomaviruses (Dela Cruz *et al.*, 2013; Wellehan *et al.*, 2011), MmelPyV1 represents the third fully described polyomavirus species in carnivorans and the first to be described in a European carnivoran.

The closest relative of MmelPyV1 is CSLPyV1 (Wellehan *et al.*, 2011). Of all known polyomavirus hosts, the sea lion is the most closely related to the European badger. Analyses using BaTS (Parker *et al.*, 2008) showed significant support for preferential clustering of polyomaviruses from more similar hosts (Table 1). Although previous analyses have ruled out strict viral-host co-divergence during polyomavirus evolution (Krumbholz *et al.*, 2009; Tao *et al.*, 2013; Warden & Lacey, 2012), this is not inconsistent with the fact that more phylogenetically related polyomaviruses are isolated from more similar hosts than expected by chance. Instead, the evolutionary history of cross-species transmission in the polyomaviruses is likely to result from the interplay of multiple factors, such as preferential host switching amongst related hosts, viral–host co-divergence and host species sympatry (e.g. Charleston & Robertson, 2002; Streicker *et al.*, 2010). Understanding the evolutionary history is complicated by the fact that some polyomaviruses have been isolated from captive animals rather than from animals living within their natural host ranges (e.g. Scuda *et al.*, 2013). It is possible that animals living in artificially close proximity may share pathogens more frequently than in the wild. Furthermore, such cross-species transmissions may be more likely to cause disease symptoms that are subsequently investigated and identified as caused by novel viruses.

Using a previously published nucleotide substitution rate for BK polyomaviruses (Firth *et al.*, 2010), we estimate that the two MmelPyV1 isolates diverged within the past few hundred years. The polyomavirus NCCR is the most variable genome region, both within and across virus species (De Gascun & Carr, 2013). The recent divergence of the French and UK isolates is supported by a deficit of genetic differences in the NCCR. The substitution rate we employed is, to our knowledge, the only published rate derived from longitudinal sampling of polyomavirus genomes. There are caveats to the rate used here. First, Firth *et al.* (2010) noted that the BK polyomavirus dataset they analysed contained comparatively weak temporal structure, hence their estimated evolutionary rate may be poorly resolved. Second, BK polyomavirus is not closely related to MmelPyV1 (especially in the late region; Fig. 5a) and the extrapolation of evolutionary rates across different viral species may lead to errors in estimates of divergence times.

Our phylogenetic analysis of all available genomes enabled us to explore the evolutionary relationships of the mammalian polyomaviruses. Four human polyomaviruses (HPyV6, HPyV7, WU and KI) were placed in different phylogenetic positions depending on the genome region analysed (Fig. 5). Although this result may be compatible with a recombinant origin for this clade, it is known that distinguishing between genetic signatures of recombination and lineage-specific variation in evolutionary rate is difficult. Tao *et al.* (2013) reported significant evolutionary rate heterogeneity between the early and late regions in the HPyV6, HPyV7, WU and KI polyomaviruses, but observed no such heterogeneity in other polyomaviruses (Tao *et al.*, 2013). The ability of such rate heterogeneity to generate apparent signatures of recombination has been noted for other viruses, including influenza virus (Worobey *et al.*, 2002). These observations, plus the observation that genetic distances and bootstrap support values are generally lower in the early region phylogeny (Fig. 5b), suggest that rate variation may be the more likely explanation for phylogenetic incongruence in the mammalian polyomaviruses.

Diagnostic PCRs to determine the presence of MmelPyV1 in different tissues suggested that the virus occurs at highest frequencies in the lymph nodes and the liver, and rarely in blood, faeces or kidney samples. In a study of primate polyomaviruses, viruses were also rarely present in these latter tissues (Scuda *et al.*, 2013). A previous metagenomic analysis of badger faeces by van den Brand *et al.* (2012) did not identify polyomavirus, supporting our suggestion that MmelPyV1 is rarely found in faeces. Future attempts to characterize MmelPyV1 or to discover new mammalian polyomaviruses should perhaps focus on solid-tissue samples rather than blood or faeces.

The known diversity of the *Polyomaviridae* has increased substantially due to increased virus discovery efforts, yet unanswered questions remain. The true host range and genomic diversity of mammalian polyomaviruses are unknown. Evolutionary rates for the family are unclear, and a molecular epidemiological investigation of polyomavirus transmission and diversity within a wild animal population has yet to be conducted. Furthermore, it is unknown why only some polyomaviruses are associated with disease and information about the aetiology of polyomaviruses is mostly limited to the viruses infecting humans. This problem is unlikely to be solved without systematic virus sampling in natural host populations, thereby avoiding sampling biases arising from the convenient sampling of easy-to-catch, high-profile or dead animals.

## Methods

### 

#### Viral discovery and sequencing of the UK isolate.

MmelPyV1 was first discovered in a cell culture supernatant, derived from the apical lobe of a badger lung in Cornwall, England, in 1996 (Banks *et al.*, 2002). The culture was established using the mink cell line NBL-7 (ATC CCL 64) by Banks *et al.* (2002). The clarified supernatant of this cell culture had been stored at −80 °C since 1997 (the ‘1997 supernatant’).

The 1997 supernatant was enriched using a sucrose cushion according to published methods (Wang *et al.*, 2006) and DNA was subsequently extracted. Randomly primed amplification (REPLI-g UltraFast reagents; Qiagen) was used to amplify all sample DNA. This method provides robust amplification of all DNA in preparation for deep sequencing, but also works particularly well at amplifying small circular templates such as the polyomavirus through rolling circle replication (reviewed by Johne *et al.*, 2009). This material was sheared using a Covaris sonicator, and standard Illumina paired-end libraries were prepared and sequenced on an Illumina MiSeq. The short read sequence data (1×10^7^, 149 nt reads) were filtered to remove low-quality reads using quasr (Watson *et al.*, 2013) and reads mapping to mink repetitive sequences.

*De novo* assembly with Velvet version 1.2.7 and VelvetOptimizer-2.2.0 was used to assemble larger sequence contigs (Zerbino, 2010; Zerbino & Birney, 2008). The resulting contigs were processed using slim, an iterative blast algorithm (Cotten *et al.*, 2014) to identify viral sequences.

The 1997 supernatant from which the MmelPyV UK isolate was discovered was recultured to increase virus stock using mink cell line NBL-7. Following lysis by freeze/thawing to release any remaining cell-associated virus, a new supernatant (henceforth called ‘MusHV1’) was produced by centrifugation. To establish whether the polyomavirus was of badger or mink cell line origin, cells from the mink cell line NBL-7 were cultured in minimum essential medium (10 % FCS/1 % penicillin/streptomycin/glutamine), and were either infected with MusHV1 or mock infected. Infection was allowed to proceed until extensive cytopathic effect of the MusHV1-infected sample. Cells and supernatant were harvested, centrifuged (1200 r.p.m., 5 min) and the cell pellet was resuspended. DNA was extracted using a Qiagen DNA Blood and Tissue kit, with lysates being passed through a QiaShredder column to reduce viscosity prior to loading onto the DNA binding columns. DNA was amplified using Invitrogen Accuprime *Taq* and primers listed in Table S4. The following reaction conditions were used: 94 °C for 5 min; 35 cycles of denaturation (94 °C, 30 s), annealing (58 °C, 30 s) and elongation (68 °C, 5 min); final elongation at 68 °C for 10 min. DNA products were resolved by agarose gel electrophoresis and visualized using ethidium bromide staining.

#### Sequencing of the French isolate.

Wild badger tissue samples were tested for the polyomavirus in an independent laboratory. Between March and May 2013, 11 badgers were captured in the Chavigny commune, France, as part of a study into a vaccine for *Mycobacterium bovis*. Following capture, badgers were maintained together in open-air pens. Ethical approval was given by ANSES/ENVA/UPEC, which is registered by the French National Working Group on Animal Ethics (CNREEA). Legal permissions were obtained from the French Ministry of Higher Education and Research (reference 00611.02) and from regional governmental committees [Direction Départementale des Territoires de Meurthe-et-Moselle, Direction Départementale de la Protection des Populations de Meurthe-et-Moselle (reference C54-431-1) and Direction Départementale de la Protection des Populations d’Indre-et-Loire (reference C 37-175-3)].

DNA was extracted from blood samples using QIAamp kits (Qiagen). Tissue samples were extracted according to published methods (Courcoul *et al.*, 2014; Hénault *et al.*, 2006). Diagnostic PCRs targeting VP1 and the NCCR were performed in eight sample types from 11 different badgers (Tables S1 and S2). A template of 250 ng organ or whole-blood DNA, or 5 µl DNA extracted from faeces, was added to the PCR master mix (AmpliTaq Gold reagents; Applied Biosystems) (primers listed in Table S4). PCRs were completed under the following conditions: 95 °C for 12 min; 45 cycles of denaturation (95 °C, 30 s), annealing (60 °C, 30 s) and elongation (72 °C, 2 min); final elongation at 72 °C for 10 min. All PCR products of expected sizes were purified and sequenced using a BigDye Terminator Cycle Sequencing kit (Applied Biosystems) on a 377 Automated DNA Sequencer (Applied Biosystems). Bases were called using Sequencing Analysis version 5.4 (Applied Biosystems) software and the resulting files analysed using Geneious version 7.1.4 (Geneious).

One of the DNA samples positive for both the NCCR and VP1 (salivary gland of badger 6) was chosen for genome amplification. Long-distance nested PCR was performed using the TaKaRa-EX PCR system (TaKaRa Bio) and the primers listed in Table S4. The amplicons were sequenced and analysed as above, producing a full-length genome. To validate the genome, independent overlapping PCRs were completed and the new amplicons resequenced (Table S4).

emboss was used to search for tandem repeats and palindromes in the NCCR (Rice *et al.*, 2000). Settings were: palindromes, minimum length 10 and 20 (one and three mismatches, respectively); tandem repeats, maximum repeat size 600, threshold score 12.

#### Phylogenetic analyses.

To construct an alignment of polyomaviruses, 63 whole-genome polyomavirus sequences were obtained from GenBank (accessed August 2014), including reference sequences from all known mammalian polyomavirus species and putative novel mammalian polyomavirus species. Avian polyomaviruses were too divergent to be aligned at the whole-genome level and were excluded. The two MmelPyV1 isolates were nearly identical, varying at 35 sites (0.67 % of the genome), so only the UK isolate was included in the phylogenetic study.

Nucleotide sequences were aligned using Muscle version 3.8.31 with default parameters (Edgar, 2004), which generated a comparatively poor-quality alignment that required manual editing using Se-Al (http://tree.bio.ed.ac.uk/software/seal/). Regions where the alignment was highly uncertain (including non-coding regions) were removed, resulting in a ‘genome-wide’ alignment of 3534 bp that covered ~70 % of the MmelPyV1 polyomavirus genome length (regions retained are shown in Fig. 2). Two further alignments were derived from the genome-wide alignment: (i) an ‘early-gene’ alignment (2034 nt) and (ii) a ‘late-gene’ alignment (1500 nt). Third codon positions were found to be significantly saturated using the test of Xia *et al.* (2003) as implemented in dambe (Xia & Lemey, 2009; Xia & Xie, 2001; Xia *et al.*, 2003) and were removed.

mega6 (Tamura *et al.*, 2013) was used to test nucleotide substitution models. For both early and late regions, the best-fit model was a general time reversible (GTR) model of nucleotide substitution, with gamma (Γ)-distributed among-site rate heterogeneity and a category of invariant sites (GTR+Γ+I). Phylogenetic trees were estimated using maximum-likelihood as implemented in Garli-2.01 (Zwickl, 2006) from the genome-wide, early-gene and late-gene alignments outlined above. Ten heuristic searches for the maximum-likelihood tree were repeated for each alignment and the tree with highest likelihood retained. One thousand maximum-likelihood bootstrap replicates were performed and the support for each node annotated onto the maximum-likelihood tree using the SumTrees functionality of DendroPy version 3.12.0 (Sukumaran & Holder, 2010).

MrBayes version 3.2.2 (Ronquist & Huelsenbeck, 2003) was used to generate a posterior sample of trees from the genome-wide alignment, using a Markov chain Monte Carlo of 3×10^6^ steps. The first 25 % of trees were discarded as ‘burn-in’. Trait values that represented the taxonomic group of the host species of each viral sequence were assigned to the phylogenetic tree tips (Table S3). The program BaTS was used (with 200 randomizations of assigned trait values performed to define a null distribution) to statistically test whether viruses found in taxonomically related hosts clustered in the virus phylogeny (Parker *et al.*, 2008).

To assess the presence of phylogenetic signal for recombination amongst polyomaviruses, the genome-wide alignment was divided into 12 overlapping subgenomic partitions. Starting from position 1, each partition was 600 nt long with a neighbouring partition overlap of 200 nt. Phylogenetic trees were estimated from each partition using the maximum-likelihood approach detailed above (except that two heuristic searches and 200 bootstrap replicates were performed for each partition). Visual inspection of these trees revealed phylogenetic inconsistencies between genome regions. Subsequently, one species was chosen randomly from every monophyletic clade that was present in all 12 partition phylogenies. Sixteen clades that were well supported in the whole-genome phylogeny were identified (15 clades supported by bootstrap support values of 100 % and one clade by 91 %). Ten species that did not belong to any clade were also included in the analysis, resulting in a reduced alignment of 26 polyomavirus species. These 26 taxa were analysed using Recco (Maydt & Lengauer, 2006) to seek evidence of recombination. Recco attempts to find the minimum cost of reconstructing each sequence in an alignment from a combination of recombination and mutation of the other sequences, where recombination and mutation have a cost penalty (Maydt & Lengauer, 2006). Probable recombination breakpoints were generated based on the minimum-cost solutions. To further investigate potential recombinants, putative recombination breakpoints were used to define new genomic partitions and maximum-likelihood phylogenies were again estimated from each partition.

To determine the divergence of the MmelPyV1 isolate genomes (UK and French), we created a whole-genome alignment containing only the two isolates plus the closest related viral species (CSLPyV1) as an outgroup. All three genome positions and almost all regions except the NCCR could be retained (4812 sites). Pairwise genetic distances were calculated using mega6 (Tamura *et al.*, 2013). The date of divergence of the UK and French MmelPyV1 isolates was estimated using previous estimates of polyomavirus substitution rates (Firth *et al.*, 2010).
